# 1,1′-{[1,1′-(Pyridinium-2,6-di­yl)diethyl­idyne]diimino}diguanidinium penta­chloridocadmate(II) monohydrate

**DOI:** 10.1107/S1600536809027196

**Published:** 2009-07-18

**Authors:** Rui-jun Xu

**Affiliations:** a Ordered Matter Science Research Center, College of Chemistry and Chemical Engineering, Southeast University, Nanjing 210096, People’s Republic of China

## Abstract

In the title organic–inorganic hybrid salt, (C_11_H_20_N_9_)[CdCl_5_]·H_2_O, the crystal structure is stabilized by intermolecular hydrogen bonds between the organic cation, the complex inorganic anion and the uncoordinated water molecule, forming a three-dimensional network.

## Related literature

For details of the synthesis, see: Valdes-Martinez *et al.* (2002 ▶).
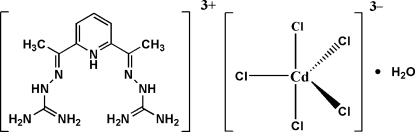


## Experimental

### 

#### Crystal data

(C_11_H_20_N_9_)[CdCl_5_]·H_2_O
*M* *_r_* = 586.03
Monoclinic, 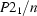

*a* = 10.638 (2) Å
*b* = 13.700 (3) Å
*c* = 14.839 (3) Å
β = 90.90 (3)°
*V* = 2162.3 (8) Å^3^
*Z* = 4
Mo *K*α radiation
μ = 1.65 mm^−1^
*T* = 298 K
0.25 × 0.20 × 0.18 mm

#### Data collection

Rigaku Mercury2 diffractometer
Absorption correction: multi-scan (*CrystalClear*; Rigaku, 2005 ▶) *T*_min_ = 0.681, *T*_max_ = 0.745
22228 measured reflections
4947 independent reflections
4155 reflections with *I* > 2σ(*I*)
*R*_int_ = 0.043

#### Refinement

*R*[*F* ^2^ > 2σ(*F* ^2^)] = 0.034
*wR*(*F* ^2^) = 0.068
*S* = 1.10
4947 reflections
246 parameters
H-atom parameters constrained
Δρ_max_ = 0.45 e Å^−3^
Δρ_min_ = −0.38 e Å^−3^

### 

Data collection: *CrystalClear* (Rigaku, 2005 ▶); cell refinement: *CrystalClear*; data reduction: *CrystalClear*; program(s) used to solve structure: *SHELXS97* (Sheldrick, 2008 ▶); program(s) used to refine structure: *SHELXL97* (Sheldrick, 2008 ▶); molecular graphics: *ORTEPIII* (Burnett & Johnson, 1996 ▶) and *ORTEP-3 for Windows* (Farrugia, 1997 ▶); software used to prepare material for publication: *PRPKAPPA* (Ferguson, 1999 ▶).

## Supplementary Material

Crystal structure: contains datablocks I, global. DOI: 10.1107/S1600536809027196/dn2466sup1.cif

Structure factors: contains datablocks I. DOI: 10.1107/S1600536809027196/dn2466Isup2.hkl

Additional supplementary materials:  crystallographic information; 3D view; checkCIF report

## Figures and Tables

**Table 1:** Hydrogen-bond geometry (Å, °)

*D*—H⋯*A*	*D*—H	H⋯*A*	*D*⋯*A*	*D*—H⋯*A*
N1—H1⋯O1*W*	0.86	2.42	3.194 (4)	151
N4—H4*A*⋯Cl5	0.86	2.31	3.154 (3)	168
N5—H5*A*⋯Cl1	0.86	2.49	3.256 (2)	149
N8—H8*B*⋯O1*W*	0.86	2.03	2.854 (3)	160
O1*W*—H1*WB*⋯Cl4	0.84	2.82	3.478 (3)	136
N3—H3⋯Cl2^i^	0.86	2.56	3.196 (2)	132
N5—H5*B*⋯Cl2^i^	0.86	2.75	3.394 (3)	133
N5—H5*B*⋯Cl4^i^	0.86	2.60	3.313 (3)	140
N7—H7⋯Cl4^ii^	0.86	2.56	3.367 (2)	156
N8—H8*A*⋯Cl1^iii^	0.86	2.40	3.247 (3)	168
N9—H9*A*⋯Cl2^iii^	0.86	2.41	3.227 (3)	160
N9—H9*B*⋯Cl4^ii^	0.86	2.67	3.450 (3)	152
O1*W*—H1*WA*⋯Cl3^iv^	0.85	2.67	3.266 (3)	129

Symmetry codes: (i) 
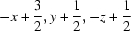
; (ii) 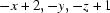; (iii) 
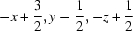
; (iv) 
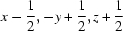
.

**Fig. 1.**
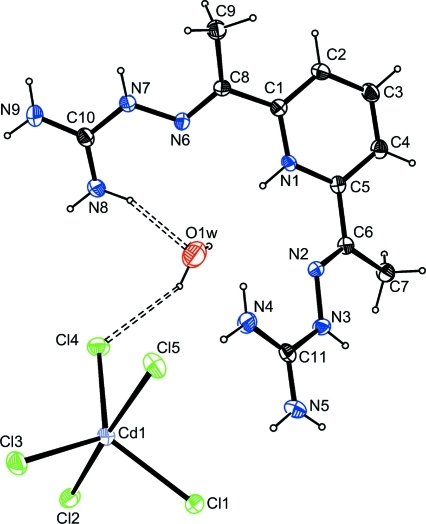
Molecular structure of the title compound,with the atom labeling sche me. Displacement ellipsoids are drawn at the 30% probability level. H atoms are represented as small spheres of arbitrary radii. H bonds are shown as dashed lines.

## supplementary crystallographic information

### Comment

The asymmetric unit of the title compound (Fig 1) consists of pentachlorocadmium, a water molecule and H_3_L, the latter resulting from protonation of the pyridyl nitrogen and the two guanyl N atoms. There are four intramolecular hydrogen bonds in the compound, *i.e.*, N4—H4···Cl5, N5—H5A···Cl1, N8—H8B···O1W and O1W—H1WB···Cl4 (table 1). The angle between the pyridine ring and the aminoguanidone moieties, N2—N3—C11—N4—N5 and N6—N7—C10—N8—N9, are 26.23 (2)° and 31.13 (1)° respectively. Additionally, there are also numerous hydrogen bonds among the terminal nitrogen atoms of the trication H_3_L, the oxygen atom of the water molecule and the chloride atoms of pentachlorocadmium anion, leading to a complex three-dimensional network.

### Experimental

The ligand *L* was prepared according to reported method (Valdes-Martinez *et al.* 2002). The title compound was prepared by refluxing an 30 ml EtOH–HCl mixture solution (v:v = 3:1) containing an equimolar amount of *L* (1.096 g, 4 mmol) and CdCl_2_ for 1 h. The resulting solution was filtered and stood still until crystals formed.

### Refinement

All H atoms attached to C atoms and N atom were fixed geometrically and treated as riding with C—H = 0.96 Å (methyl) or 0.93 Å (aromatic) and N—H = 0.86 Å with U_iso_(H) = 1.2U_eq_(Caromatic or N) and U_iso_(H) = 1.5U_eq_(Cmethyl). H atoms of water molecule were located in difference Fourier maps and included in the subsequent refinement using restraints (O-H= 0.85 (1)Å and H···H= 1.39 (2)Å) with U_iso_(H) = 1.5U_eq_(O). In the last stage of structure refinement, they were treated as riding on their parent O atom.

### Crystal data

**Table:**

(C_11_H_20_N_9_)[CdCl_5_]·H_2_O	*F*(000) = 1168
*M**_r_* = 586.03	*D*_x_ = 1.800 Mg m^−^^3^
Monoclinic, *P*2_1_/*n*	Mo *K*α radiation, λ = 0.71073 Å
Hall symbol: -P 2yn	Cell parameters from 19919 reflections
*a* = 10.638 (2) Å	θ = 3.1–27.6°
*b* = 13.700 (3) Å	µ = 1.65 mm^−^^1^
*c* = 14.839 (3) Å	*T* = 298 K
β = 90.90 (3)°	Prism, colourless
*V* = 2162.3 (8) Å^3^	0.25 × 0.20 × 0.18 mm
*Z* = 4	

### Data collection

**Table:**

Rigaku Mercury2 (2× 2 bin mode) diffractometer	4947 independent reflections
Radiation source: fine-focus sealed tube	4155 reflections with *I* > 2σ(*I*)
graphite	*R*_int_ = 0.043
Detector resolution: 13.6612 pixels mm^-1^	θ_max_ = 27.5°, θ_min_ = 3.1°
CCD_Profile_fitting scans	*h* = −13→13
Absorption correction: multi-scan (*CrystalClear*; Rigaku, 2005)	*k* = −17→17
*T*_min_ = 0.681, *T*_max_ = 0.745	*l* = −19→19
22228 measured reflections	

### Refinement

**Table:**

Refinement on *F*^2^	Primary atom site location: structure-invariant direct methods
Least-squares matrix: full	Secondary atom site location: difference Fourier map
*R*[*F*^2^ > 2σ(*F*^2^)] = 0.034	Hydrogen site location: inferred from neighbouring sites
*wR*(*F*^2^) = 0.068	H-atom parameters constrained
*S* = 1.10	*w* = 1/[σ^2^(*F*_o_^2^) + (0.0222*P*)^2^ + 1.3165*P*] where *P* = (*F*_o_^2^ + 2*F*_c_^2^)/3
4947 reflections	(Δ/σ)_max_ = 0.032
246 parameters	Δρ_max_ = 0.45 e Å^−^^3^
0 restraints	Δρ_min_ = −0.38 e Å^−^^3^

### Special details

**Table:**

Geometry. All esds (except the esd in the dihedral angle between two l.s. planes) are estimated using the full covariance matrix. The cell esds are taken into account individually in the estimation of esds in distances, angles and torsion angles; correlations between esds in cell parameters are only used when they are defined by crystal symmetry. An approximate (isotropic) treatment of cell esds is used for estimating esds involving l.s. planes.
Refinement. Refinement of *F*^2^ against ALL reflections. The weighted *R*-factor *wR* and goodness of fit *S* are based on *F*^2^, conventional *R*-factors *R* are based on *F*, with *F* set to zero for negative *F*^2^. The threshold expression of *F*^2^ > σ(*F*^2^) is used only for calculating *R*-factors(gt) *etc*. and is not relevant to the choice of reflections for refinement. *R*-factors based on *F*^2^ are statistically about twice as large as those based on *F*, and *R*- factors based on ALL data will be even larger.

### Fractional atomic coordinates and isotropic or equivalent isotropic displacement parameters (Å^2^)

**Table:**

	*x*	*y*	*z*	*U*_iso_*/*U*_eq_	
Cd1	0.91113 (2)	0.240737 (14)	0.143910 (13)	0.03119 (7)	
Cl1	0.88183 (8)	0.41966 (5)	0.11166 (5)	0.04173 (18)	
Cl2	0.68199 (7)	0.21144 (5)	0.06726 (5)	0.03978 (18)	
Cl3	1.02297 (8)	0.14273 (6)	0.03080 (5)	0.0464 (2)	
Cl4	0.82906 (7)	0.15219 (6)	0.28367 (5)	0.04094 (18)	
Cl5	1.11659 (7)	0.27884 (6)	0.23687 (5)	0.04219 (19)	
N1	0.89827 (19)	0.31005 (15)	0.68390 (14)	0.0238 (5)	
H1	0.8938	0.2862	0.6304	0.029*	
N2	0.8635 (2)	0.43124 (15)	0.54498 (14)	0.0252 (5)	
N3	0.8349 (2)	0.48151 (16)	0.46731 (14)	0.0302 (5)	
H3	0.7842	0.5304	0.4672	0.036*	
N4	0.9582 (3)	0.37103 (17)	0.39331 (16)	0.0428 (6)	
H4A	0.9939	0.3508	0.3452	0.051*	
H4B	0.9673	0.3390	0.4428	0.051*	
N5	0.8730 (2)	0.50235 (18)	0.31717 (15)	0.0354 (6)	
H5A	0.9073	0.4842	0.2678	0.042*	
H5B	0.8276	0.5543	0.3182	0.042*	
N6	0.9327 (2)	0.11265 (15)	0.66384 (14)	0.0275 (5)	
N7	0.9631 (2)	0.01760 (16)	0.64548 (15)	0.0334 (5)	
H7	1.0230	−0.0113	0.6745	0.040*	
N8	0.8182 (2)	0.01826 (18)	0.52850 (16)	0.0396 (6)	
H8A	0.7755	−0.0119	0.4875	0.048*	
H8B	0.8094	0.0802	0.5353	0.048*	
N9	0.9142 (3)	−0.12433 (18)	0.57366 (18)	0.0488 (7)	
H9A	0.8735	−0.1574	0.5336	0.059*	
H9B	0.9668	−0.1531	0.6094	0.059*	
C1	0.9503 (2)	0.25487 (18)	0.74947 (17)	0.0248 (5)	
C2	0.9574 (3)	0.2929 (2)	0.83576 (18)	0.0325 (6)	
H2	0.9943	0.2568	0.8822	0.039*	
C3	0.9094 (3)	0.3846 (2)	0.85289 (18)	0.0356 (7)	
H3A	0.9125	0.4096	0.9112	0.043*	
C4	0.8568 (3)	0.4394 (2)	0.78360 (17)	0.0306 (6)	
H4	0.8246	0.5013	0.7949	0.037*	
C5	0.8526 (2)	0.40094 (18)	0.69727 (17)	0.0234 (5)	
C6	0.8104 (2)	0.45750 (18)	0.61734 (17)	0.0252 (6)	
C7	0.7170 (3)	0.5381 (2)	0.62813 (19)	0.0353 (7)	
H7A	0.7600	0.5968	0.6460	0.053*	
H7B	0.6577	0.5204	0.6734	0.053*	
H7C	0.6733	0.5488	0.5719	0.053*	
C8	0.9938 (2)	0.15541 (18)	0.72746 (17)	0.0243 (5)	
C9	1.1001 (3)	0.1131 (2)	0.7819 (2)	0.0387 (7)	
H9C	1.0674	0.0701	0.8269	0.058*	
H9D	1.1464	0.1649	0.8107	0.058*	
H9E	1.1547	0.0773	0.7430	0.058*	
C10	0.8962 (3)	−0.0298 (2)	0.58035 (18)	0.0315 (6)	
C11	0.8901 (3)	0.4506 (2)	0.39114 (18)	0.0296 (6)	
O1W	0.7726 (3)	0.22202 (18)	0.5047 (2)	0.0695 (8)	
H1WA	0.7156	0.2329	0.5427	0.104*	
H1WB	0.7448	0.2212	0.4511	0.104*	

### Atomic displacement parameters (Å^2^)

**Table:**

	*U*^11^	*U*^22^	*U*^33^	*U*^12^	*U*^13^	*U*^23^
Cd1	0.03641 (12)	0.02847 (12)	0.02866 (11)	0.00307 (9)	−0.00053 (8)	−0.00175 (8)
Cl1	0.0584 (5)	0.0293 (4)	0.0371 (4)	0.0015 (3)	−0.0135 (3)	−0.0001 (3)
Cl2	0.0407 (4)	0.0323 (4)	0.0460 (4)	−0.0014 (3)	−0.0088 (3)	0.0055 (3)
Cl3	0.0526 (5)	0.0486 (5)	0.0379 (4)	0.0167 (4)	−0.0043 (4)	−0.0125 (3)
Cl4	0.0444 (4)	0.0433 (4)	0.0351 (4)	−0.0054 (3)	−0.0023 (3)	0.0066 (3)
Cl5	0.0293 (4)	0.0566 (5)	0.0405 (4)	0.0036 (3)	−0.0015 (3)	−0.0156 (3)
N1	0.0263 (12)	0.0259 (11)	0.0192 (10)	−0.0011 (9)	0.0021 (9)	−0.0031 (8)
N2	0.0288 (12)	0.0231 (11)	0.0236 (11)	0.0011 (9)	0.0001 (9)	0.0007 (9)
N3	0.0350 (13)	0.0292 (12)	0.0264 (12)	0.0093 (10)	0.0007 (10)	0.0007 (9)
N4	0.0649 (18)	0.0338 (14)	0.0300 (13)	0.0124 (13)	0.0137 (12)	−0.0029 (10)
N5	0.0345 (14)	0.0473 (15)	0.0243 (12)	0.0015 (11)	0.0008 (10)	0.0007 (10)
N6	0.0329 (13)	0.0228 (11)	0.0269 (12)	0.0018 (9)	0.0009 (10)	−0.0020 (9)
N7	0.0373 (14)	0.0295 (12)	0.0332 (13)	0.0073 (10)	−0.0095 (11)	−0.0055 (10)
N8	0.0488 (16)	0.0329 (13)	0.0366 (14)	−0.0056 (12)	−0.0141 (12)	−0.0023 (11)
N9	0.0656 (19)	0.0311 (14)	0.0492 (16)	0.0037 (13)	−0.0145 (14)	−0.0112 (12)
C1	0.0216 (13)	0.0262 (13)	0.0265 (13)	−0.0032 (10)	0.0022 (10)	0.0007 (10)
C2	0.0357 (16)	0.0355 (15)	0.0263 (14)	−0.0001 (13)	−0.0041 (12)	0.0013 (12)
C3	0.0441 (18)	0.0384 (17)	0.0244 (14)	−0.0012 (13)	0.0018 (13)	−0.0063 (12)
C4	0.0327 (15)	0.0305 (15)	0.0288 (14)	0.0007 (12)	0.0063 (12)	−0.0044 (11)
C5	0.0201 (13)	0.0252 (13)	0.0250 (13)	−0.0026 (10)	0.0057 (10)	−0.0025 (10)
C6	0.0221 (13)	0.0263 (14)	0.0272 (14)	0.0003 (10)	0.0028 (11)	−0.0020 (10)
C7	0.0339 (16)	0.0368 (16)	0.0350 (16)	0.0122 (13)	−0.0005 (13)	−0.0068 (12)
C8	0.0237 (13)	0.0265 (14)	0.0228 (13)	−0.0003 (10)	0.0021 (11)	0.0014 (10)
C9	0.0335 (17)	0.0387 (17)	0.0436 (17)	0.0074 (13)	−0.0108 (14)	−0.0069 (13)
C10	0.0363 (16)	0.0318 (15)	0.0265 (14)	−0.0038 (12)	0.0028 (12)	−0.0032 (11)
C11	0.0303 (15)	0.0324 (15)	0.0260 (14)	−0.0064 (12)	−0.0016 (11)	−0.0056 (11)
O1W	0.0682 (18)	0.0488 (15)	0.091 (2)	0.0023 (13)	−0.0178 (16)	0.0015 (14)

### Geometric parameters (Å, °)

**Table:**

Cd1—Cl3	2.4695 (9)	N8—H8B	0.8600
Cd1—Cl1	2.5160 (9)	N9—C10	1.313 (4)
Cd1—Cl4	2.5675 (9)	N9—H9A	0.8600
Cd1—Cl5	2.6188 (10)	N9—H9B	0.8600
Cd1—Cl2	2.7036 (10)	C1—C2	1.383 (4)
N1—C1	1.344 (3)	C1—C8	1.477 (3)
N1—C5	1.352 (3)	C2—C3	1.381 (4)
N1—H1	0.8600	C2—H2	0.9300
N2—C6	1.273 (3)	C3—C4	1.385 (4)
N2—N3	1.373 (3)	C3—H3A	0.9300
N3—C11	1.350 (3)	C4—C5	1.385 (3)
N3—H3	0.8600	C4—H4	0.9300
N4—C11	1.309 (4)	C5—C6	1.481 (3)
N4—H4A	0.8600	C6—C7	1.496 (4)
N4—H4B	0.8600	C7—H7A	0.9600
N5—C11	1.317 (3)	C7—H7B	0.9600
N5—H5A	0.8600	C7—H7C	0.9600
N5—H5B	0.8600	C8—C9	1.496 (4)
N6—C8	1.280 (3)	C9—H9C	0.9600
N6—N7	1.370 (3)	C9—H9D	0.9600
N7—C10	1.356 (3)	C9—H9E	0.9600
N7—H7	0.8600	O1W—H1WA	0.8473
N8—C10	1.301 (4)	O1W—H1WB	0.8446
N8—H8A	0.8600		
			
Cl3—Cd1—Cl1	117.39 (3)	C3—C2—H2	120.1
Cl3—Cd1—Cl4	117.76 (3)	C1—C2—H2	120.1
Cl1—Cd1—Cl4	124.84 (3)	C2—C3—C4	120.2 (3)
Cl3—Cd1—Cl5	93.40 (3)	C2—C3—H3A	119.9
Cl1—Cd1—Cl5	90.34 (3)	C4—C3—H3A	119.9
Cl4—Cd1—Cl5	87.73 (3)	C3—C4—C5	119.1 (3)
Cl3—Cd1—Cl2	94.25 (3)	C3—C4—H4	120.4
Cl1—Cd1—Cl2	87.50 (3)	C5—C4—H4	120.4
Cl4—Cd1—Cl2	87.36 (3)	N1—C5—C4	118.7 (2)
Cl5—Cd1—Cl2	172.19 (2)	N1—C5—C6	118.0 (2)
C1—N1—C5	123.8 (2)	C4—C5—C6	123.1 (2)
C1—N1—H1	118.1	N2—C6—C5	113.2 (2)
C5—N1—H1	118.1	N2—C6—C7	127.1 (2)
C6—N2—N3	118.1 (2)	C5—C6—C7	119.6 (2)
C11—N3—N2	116.9 (2)	C6—C7—H7A	109.5
C11—N3—H3	121.6	C6—C7—H7B	109.5
N2—N3—H3	121.6	H7A—C7—H7B	109.5
C11—N4—H4A	120.0	C6—C7—H7C	109.5
C11—N4—H4B	120.0	H7A—C7—H7C	109.5
H4A—N4—H4B	120.0	H7B—C7—H7C	109.5
C11—N5—H5A	120.0	N6—C8—C1	115.3 (2)
C11—N5—H5B	120.0	N6—C8—C9	126.3 (2)
H5A—N5—H5B	120.0	C1—C8—C9	118.3 (2)
C8—N6—N7	117.6 (2)	C8—C9—H9C	109.5
C10—N7—N6	118.3 (2)	C8—C9—H9D	109.5
C10—N7—H7	120.9	H9C—C9—H9D	109.5
N6—N7—H7	120.9	C8—C9—H9E	109.5
C10—N8—H8A	120.0	H9C—C9—H9E	109.5
C10—N8—H8B	120.0	H9D—C9—H9E	109.5
H8A—N8—H8B	120.0	N8—C10—N9	123.1 (3)
C10—N9—H9A	120.0	N8—C10—N7	120.1 (3)
C10—N9—H9B	120.0	N9—C10—N7	116.8 (3)
H9A—N9—H9B	120.0	N4—C11—N5	122.5 (3)
N1—C1—C2	118.3 (2)	N4—C11—N3	119.3 (2)
N1—C1—C8	119.0 (2)	N5—C11—N3	118.2 (3)
C2—C1—C8	122.6 (2)	H1WA—O1W—H1WB	112.7
C3—C2—C1	119.9 (3)		

### Hydrogen-bond geometry (Å, °)

**Table:**

*D*—H···*A*	*D*—H	H···*A*	*D*···*A*	*D*—H···*A*
N1—H1···O1W	0.86	2.42	3.194 (4)	151
N4—H4A···Cl5	0.86	2.31	3.154 (3)	168
N5—H5A···Cl1	0.86	2.49	3.256 (2)	149
N8—H8B···O1W	0.86	2.03	2.854 (3)	160
O1W—H1WB···Cl4	0.84	2.82	3.478 (3)	136
N3—H3···Cl2^i^	0.86	2.56	3.196 (2)	132
N5—H5B···Cl2^i^	0.86	2.75	3.394 (3)	133
N5—H5B···Cl4^i^	0.86	2.60	3.313 (3)	140
N7—H7···Cl4^ii^	0.86	2.56	3.367 (2)	156
N8—H8A···Cl1^iii^	0.86	2.40	3.247 (3)	168
N9—H9A···Cl2^iii^	0.86	2.41	3.227 (3)	160
N9—H9B···Cl4^ii^	0.86	2.67	3.450 (3)	152
O1W—H1WA···Cl3^iv^	0.85	2.67	3.266 (3)	129

Symmetry codes: (i) −*x*+3/2, *y*+1/2, −*z*+1/2; (ii) −*x*+2, −*y*, −*z*+1; (iii) −*x*+3/2, *y*−1/2, −*z*+1/2; (iv) *x*−1/2, −*y*+1/2, *z*+1/2.

## References

[bb1] Burnett, M. N. & Johnson, C. K. (1996). *ORTEPIII* Report ORNL-6895. Oak Ridge National Laboratory, Tennessee, USA.

[bb2] Farrugia, L. J. (1997). *J. Appl. Cryst.* **30**, 565.

[bb3] Ferguson, G. (1999). *PRPKAPPA* University of Guelph, Canada.

[bb4] Rigaku (2005). *CrystalClear* Rigaku Corporation, Tokyo, Japan.

[bb5] Sheldrick, G. M. (2008). *Acta Cryst.* A**64**, 112–122. 10.1107/S0108767307043930 18156677

[bb6] Valdes-Martinez, J., Alstrum-Acevedo, J. H., Toscano, R. A., Hernandez-Ortega, S., Espinosa-Perez, G., West, X. D. & Helfrich, B. (2002). *Polyhedron*, **21**, 409–416.

